# Acupuncture for treating alopecia areata: A systematic review and meta-analysis

**DOI:** 10.1097/MD.0000000000048073

**Published:** 2026-03-20

**Authors:** Ji Hee Jun, Hye Won Lee, Tae-Young Choi, Myeong Soo Lee

**Affiliations:** a KM Science Research Division, Korea Institute of Oriental Medicine, Daejeon, Republic of Korea; b KM Convergence Research Division, Korea Institute of Oriental Medicine, Daejeon, Republic of Korea.

**Keywords:** acupuncture, alopecia areata, meta-analysis, systematic review

## Abstract

**Background:**

Alopecia areata (AA) is characterized by hair loss triggered by an autoimmune response that leads to emotional distress and a poor quality of life. However, conventional treatments may also have potential side effects. Therefore, traditional medicine methods such as acupuncture are emerging as alternative treatments for AA, with recent evidence suggesting their effectiveness and prompting further research to assess their safety and efficacy.

**Methods:**

This systematic review aimed to evaluate the current evidence regarding AA treatment using acupuncture. We searched 13 databases until December 2024 for studies that compared the efficacy of acupuncture and Western medicine in treating AA. The present review solely targeted randomized controlled trials (RCTs). The risk of bias was assessed using the Risk of Bias tool (ROB), whereas the quality of evidence was evaluated using the Grading of Recommendations, Assessment, Development, and Evaluation approach.

**Results:**

Eleven RCTs with 1144 participants were included in this review. The included studies had an unclear risk of bias in most domains. Among these, 7 RCTs tested the total treatment effects of combining plum-blossom needling with western medicine compared with western medicine alone. The results revealed that combining plum-blossom needling with western medicine resulted in a higher rate of improved symptoms than using western medicine alone (RR 1.09, 95% CI 1.03–1.16, *I*^2^ = 50%, *P* = .005, 7 RCTs, 756 participants, low certainty of evidence).

**Conclusion:**

This review provides evidence for the benefits of acupuncture in AA treatment. However, the quality of the evidence was low, and the sample size was small. Therefore, future large-scale studies should be conducted using more rigorous methodologies.

## 1. Introduction

Alopecia areata (AA) is a hair loss disorder caused by an autoimmune reaction that causes emotional distress and reduces the quality of life (QoL). Its prevalence is estimated to be approximately 2% in the global population.^[1,2]^ Currently, the Food and Drug Administration has approved 2 treatments for AA: minoxidil and finasteride.^[3,4]^ Minoxidil acts through multiple pathways to promote hair growth, whereas finasteride inhibits 5α-reductase type II isoenzyme. Oral minoxidil predominantly causes hypertrichosis and cardiovascular system (CVS) symptoms/signs in a dose-dependent manner, whereas oral finasteride and dutasteride are associated with sexual dysfunction and neuropsychiatric side effects.^[5–8]^ Topical and injectable treatments are available for AA; however, these have variable results and do not provide effective treatment or long-term safety.^[9]^ Consequently, traditional medicine techniques such as acupuncture, pharmacopuncture,^[10]^ microneedling,^[11,12]^ and needle embedding^[13]^ have recently emerged as treatment methods for AA. Despite the array of available treatment options, these methods produce variable outcomes, and no current treatment can induce and sustain AA remission.

Acupuncture has been a long-term treatment method used in East Asia for several years. Recent evidence suggests that acupuncture may be an effective treatment for AA.^[14]^ Plum-blossom needling (PBN) is a dermal needling technique that employs 5 or 7 needle tips at the top. PBN tapping is an external treatment method with a long history of use in traditional medicine. It involves tapping the body surface and meridians with the PBN to treat various diseases including AA and skin disorders.^[15]^ The exact cause of AA in Western medicine remains unknown; however, studies have shown that acupuncture can reduce hair loss by minimizing inflammatory attacks on the hair bulb.^[16]^ Additionally, acupuncture may stimulate hair follicles, improve blood circulation, and enhance local collaterals. Increased blood flow and nutrient delivery contribute to hair growth. Moreover, reducing stress and anxiety can positively affect growth.

Previous reviews^[17–23]^ have reported that acupuncture and western medicine are used to treat AA. Moreover, previous reviews have only searched Chinese databases, and no systematic review has reported the effectiveness of acupuncture alone or acupuncture combined with Western medicine in treating AA. Therefore, this review aimed to update the current evidence and assess the safety and efficacy of acupuncture in AA treatment. This study included acupuncture combined with other traditional medical therapies for AA. The findings of this review are expected to validate the use of acupuncture as an intervention to achieve better treatment effects in patients with AA.

## 2. Methods

### 2.1. Study registration and protocol

This study was published in Journal^[24]^ (Supplementary 1, Supplemental Digital Content, https://links.lww.com/MD/R556) and was registered with PROSPERO (registration number: CRD42015020397). This review was presented according to the Preferred Reporting Items for Systematic Reviews and Meta-analyses (PRISMA) 2020 statement checklist.^[25]^ Ethical approval was not required for this study because it was a systematic review and meta-analysis of previously published literature.

### 2.2. Search strategy

Thirteen databases were searched until December 2024: PubMed, Cochrane Library, AMED, EMBASE, China National Knowledge Infrastructure, Wanfang, VIP, DBpia, Korean Studies Information Service System (KISS), KoreaMed, Korea Science, the National Library of Korea, and RISS. We also searched for relevant journals and the Google Scholar database. The search was conducted in English, Chinese, and Korean languages. The search terms were “AA” and “acupuncture.” Details of the search strategies are provided in Supplementary 2, Supplemental Digital Content, https://links.lww.com/MD/R556.

### 2.3. Inclusion and exclusion criteria

#### 2.3.1. Type of studies

Randomized controlled trials (RCTs) and quasi-RCTs were included. Non-RCTs, case studies, reviews, dissertations, and animal studies were excluded.

#### 2.3.2. Type of participants

Participants of all sexes and ages diagnosed with AA were included.

#### 2.3.3. Types of intervention

All acupuncture treatments (acupuncture, PBN, and fire needle therapy) were performed. The control group received western medicine, usual care, no treatment, sham acupuncture, or other complementary therapies. Studies that compared the 2 types of acupuncture were excluded.

#### 2.3.4. Types of outcome measurements

The primary outcomes included the total treatment effect, hair regrowth, and rate of hair loss. The secondary outcome included the Quality of life (QoL), satisfaction with hair appearance, adverse events (AEs).

### 2.4. Data collection, extraction, and assessment

#### 2.4.1. Study selection and data extraction

Two authors (JHJ and HYL) screened the titles and abstracts of the selected studies and subsequently retrieved and reviewed the full texts of the eligible studies for final inclusion. If there was no consensus, a third author (MSL) decided on study selection. The screening and selection processes were conducted using a PRISMA flow diagram. Data on the first author, sample size, age, diagnostic criteria, intervention group, control group, primary outcome, results, and AEs were extracted from the collected information. Data extraction was conducted using the JHJ software. If data were unavailable, the authors of the included studies were contacted for further clarification.

#### 2.4.2. Assessment of risk of bias

The authors (JHJ and TYC) assessed the risk of bias^[26,27]^ using the Cochrane Handbook for Systematic Review of Interventions. Any disagreements were resolved by consensus reached through discussions among the authors. The following 7 domains were assessed: sequence generation, allocation concealment, participant anonymization, outcome assessors, incomplete outcome data, selective outcome reporting, and other biases. The domains were judged as “Low,” “High,” or “Uncertain.”

#### 2.4.3. Quality of evidence assessment

Grading of Recommendations, Assessment, Development, and Evaluation (GRADE) tool (https://www.gradepro.org/). The assessment involved the following 5 domains: ROB, inconsistency, indirectness, imprecision of the synthesized results, and publication bias. The quality of the evidence was classified as “Very low,” “Low,” “Moderate,” or “High.”

#### 2.4.4. Data analysis

Data analysis was performed using RevMan software (Ver 5.4). We used the total treatment effect as the risk ratio with 95% confidence intervals for dichotomous data. We used the mean difference (MD) with a 95% CI for continuous data. The standardized MD (SMD) outcome variable with different scales was used instead of weighted MD (WMD). Chi-square and Higgins *I*^2^ tests were used to assess heterogeneity.^[27]^ A fixed-effects model was used if heterogeneity was not evident, whereas a random-effects model was used if heterogeneity was evident. Subgroups were administered therapy based on the type of control group. Publication bias was assessed using a funnel plot, whenever possible.

## 3. Results

### 3.1. Search results

**Figure 1.**
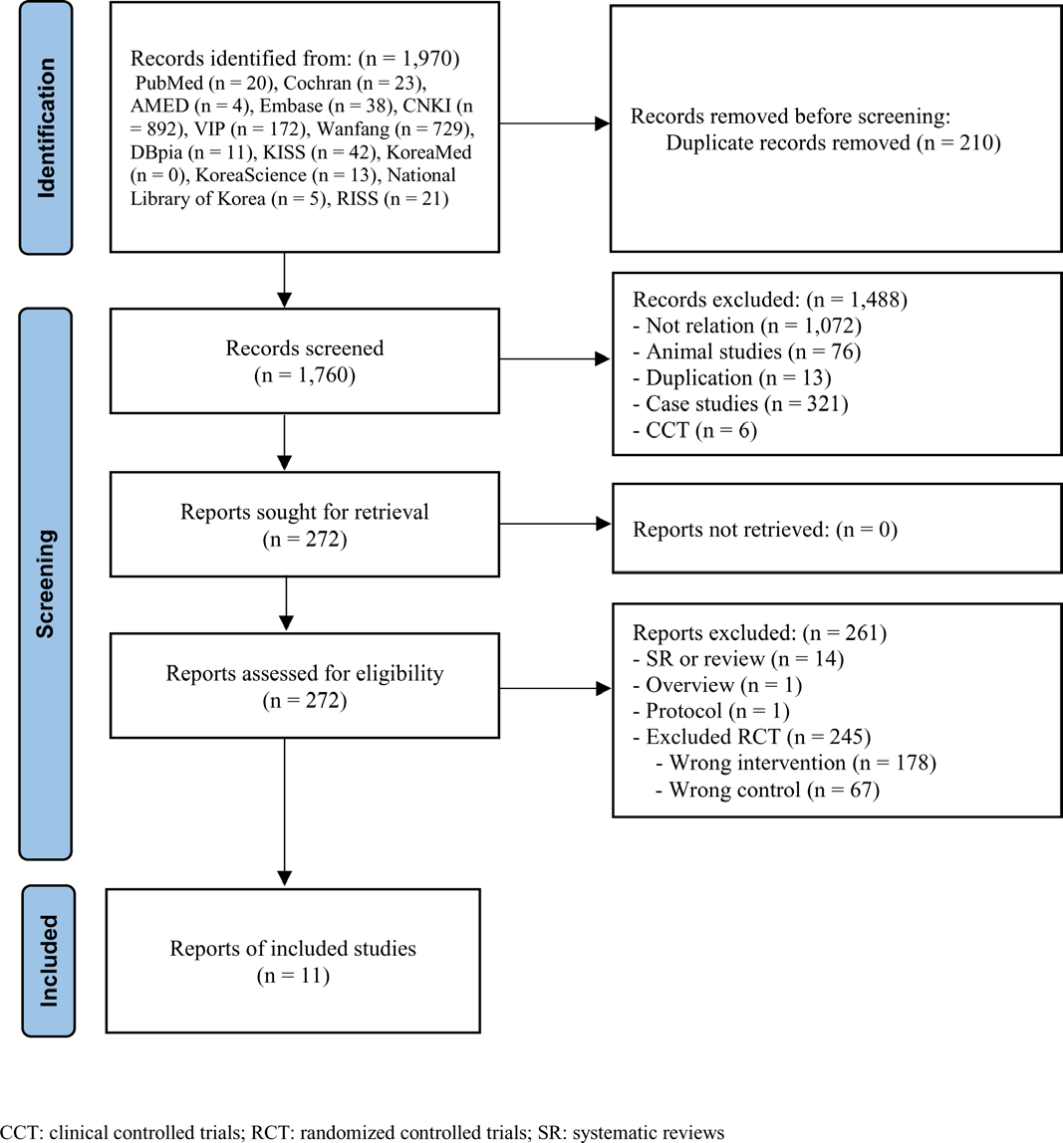
Flow chart of the trial selection process. CCT = clinical controlled trials, RCT = randomized controlled trials, SR = systematic reviews.

A search of the 13 electronic databases yielded 1970 studies. We excluded 210 studies due to duplication and evaluated 1760 studies. Eleven RCTs^[28–38]^ were included in this review after assessing their titles, abstracts, and full text (Fig. 1). Table 1 summarizes the included RCTs.

**Table 1:** Summary of randomized controlled trials of acupuncture for Alopecia.

The first author (yr) [ref]	Sample size (M/F); age, mean	Diagnostic criteria	Intervention group	Control group	Main outcomes	Result	Adverse events
Zhang (2020a)	87 (52/ 35) (A) 33; (B) 30; (C) 34	n.r.	(A) AT (n.r., once a week for 8 weeks, n = 33)	(B) External (Minoxidil Tincture, 3~5 min, n.r., 8 weeks, n = 24) (C) Intralesional injection (Triamcinolone acetonide, once every 3 weeks for 8 weeks), n = 30)	Total treatment effect	A versus B: RR 1.04 [0.86, 1.25], NS A versus C: RR 0.94 [0.83, 1.07], NS	Folliculitis (A:1); skin atrophy (C:8); male facial acne (C:2); furuncle (C:1); irregular menses (C:1); pilosity (B:1)
Xu (2015)	60 (30/ 30) (A) 25; (B) 27	《Guiding principles for clinical research on new Chinese medicines》1997	(A) AT (20 min, once daily for 4 weeks) + PBN (2~3 min, 3 times a week for 4 weeks, n = 30)	(B) External (Minoxidil Tincture, 2 times a day for 4 weeks, n = 30)	Total treatment effect	RR 1.50 [1.09, 2.06], *P = .01*	n.r.
Mai (2018)	112 (62/ 50) (A) 29; (B) 30; (C) 31	n.r.	(A) PBN (3~5 min, 2 times a week for 12 weeks, n = 32) (C) A plus B, n = 50	(B) External (Minoxidil Tincture, 2 times a day for 12 weeks, n = 30)	Total treatment effect	A versus B: RR 1.08 [0.71, 1.64], NS; C versus B: RR 1.23 [0.89, 1.69], NS	Pain (A: 25, C: 42)
Zhang (2020b)	68 (37/ 31) (A) 25; (B) 26	《Dermatology》 《Traditional Chinese Medicine Surgery》	(A) PBN (n.r., 3 times a week for 12 weeks, n = 34), plus B	(B) External (Minoxidil Tincture, n.r., 12 weeks, n = 34)	Total treatment effect	RR 1.33 [0.98, 1.81], NS	Mild pain (A:3; B:2); slight erythema on local skin (A:1; B:2)
Luo (2015)	82 (48/ 34) 44	n.r.	(A) PBN (8~10 min, once over 3 weeks for 6 weeks, n = 42), plus B	(B) Intralesional injection (Compound Betamethasone, once over 3 weeks for 6 weeks, n = 40)	Total treatment effect	RR 1.08 [0.97, 1.22], NS	Atrophoderma (B:7); Cushing syndrome (B:1); folliculitis (B:4); hiccup (B:3); sleep disorders (B:3)
Zhang (2014)	64 (41/ 23) (A) 33; (B) 30	n.r.	(A) PBN (5 min, once a week for 12 weeks, n = 32), plus B	(B) Intralesional injection (Triamcinolone acetonide, once a week for 12 weeks, n = 32)	1) Total treatment effect 2) Satisfaction degree	1) RR 1.07 [0.96, 1.19], NS 2) RR 1.13 [0.94, 1.36], NS	Atrophoderma (A:2, B:4); bruise (A:19, B:15); folliculitis (A:3, B:2); local pain (A:32, B:31); pigmentation (A:2, B:2)
Xu (2023)	80 (35/ 45) (A) 30; (B) 30	《Dermatology》	(A) PBN (5 min, 2 times a week for 12 weeks, n = 40), plus B	(B) Intralesional injection (Compound Betamethasone, once over 4 weeks for 12 weeks, n = 40)	1) Total treatment effect 2) QoL (DLQI)	1) RR 1.05 [0.95, 1.17], NS 2) MD −2.64 [-3.33, −1.95], *P < .00001*	Skin atrophy (A:6, B:5); pigmentation (A:3, B:4); folliculitis (A:1, B:2)
Zhang (2021)	287 (175/ 112) (A) 38; (B) 38	《Guidelines for diagnosis and treatment of alopecia areata in China 》	(A) PBN (n.r., once over 2 weeks) + External (Triamcinolone acetonide, once over 2 weeks for 12 weeks), n = 155	(B) Intralesional injection (Triamcinolone acetonide, once over 2 weeks for 12 weeks), n = 132)	Total treatment effect	RR 1.17 [1.07, 1.27], *P* = *.0005*	Abdominal distension (B:2); pruritus (A:2, B:4); erythema (A:1, B:3); scorching hot (A:1, B:6)
Tang (2008)	110 (42/ 68) 31	Chinese Society of Integrated Chinese and Western Medicine Dermatology and Venereology 《Diagnosis and curative effect judgment criteria of 5 skin diseases with integrated Traditional Chinese and Western Medicine》	(A) PBN (n.r., once over 4 weeks) + External (Compound Betamethasone, once over 4 weeks for 8 weeks, n = 55)	(B) Intralesional injection (Triamcinolone acetonide, once over 4 weeks for 8 weeks, n = 55)	Total treatment effect	RR 1.04 [0.98, 1.10], NS	Local pain (B:1)
Wu (2017)	80 (41/ 39) (A) 35; (B) 36	《Dermatology》	(A) Fire needle (n.r., once a week for 12 weeks, n = 40), plus B	(B) External (Minoxidil Tincture, once a week for 12 weeks, n = 40)	Total treatment effect	RR 1.05 [0.97, 1.15], NS	Mild redness and itching (A:3; B:2)
Xie (2021)	114 (67/ 47) (A) 43; (B) 43	《Dermatology》	(A) Fire needle (n.r., once a week for 12 weeks, n = 57), plus B	(B) External (Halometasone Cream, once a week for 12 weeks, n = 57)	1) Total treatment effect 2) Hair growth time	1) RR 1.15 [1.00, 1.31], *P = .04* 2) MD −4.62 [-6.23, −3.01], *P < .00001*	Mild folliculitis (A:2; B:1)

Italic values indicate statistically significant results.

AT = acupuncture, DLQI = dermatology quality of life index, EA = electroacupuncture, n.r. = not reported, PBN = plum-blossom needle, SC = Subcutaneous, WM = western medicine.

### 3.2. Characteristics of the included studies

All trials^[28–38]^ were conducted and published in China. The number of participants and treatment duration ranged from 60 to 287 and from 4 to 12 weeks, respectively. The intervention group included 3 types of acupuncture: usual acupuncture,^[28]^ usual acupuncture plus PBN,^[29]^ PBN,^[30–36]^ and fire needling.^[37,38]^ The control group received western medicine (injection^[28,31–36]^ and external^[28–31,37,38]^). Nine RCTs^[29,31–38]^ used a 2-arm parallel group design and 2 RCTs^[28,30]^ used a 3-arm parallel group. The included studies used total treatment effect as the outcome measure for the intervention and control groups.

### 3.3. Risk of bias

**Figure 2.**
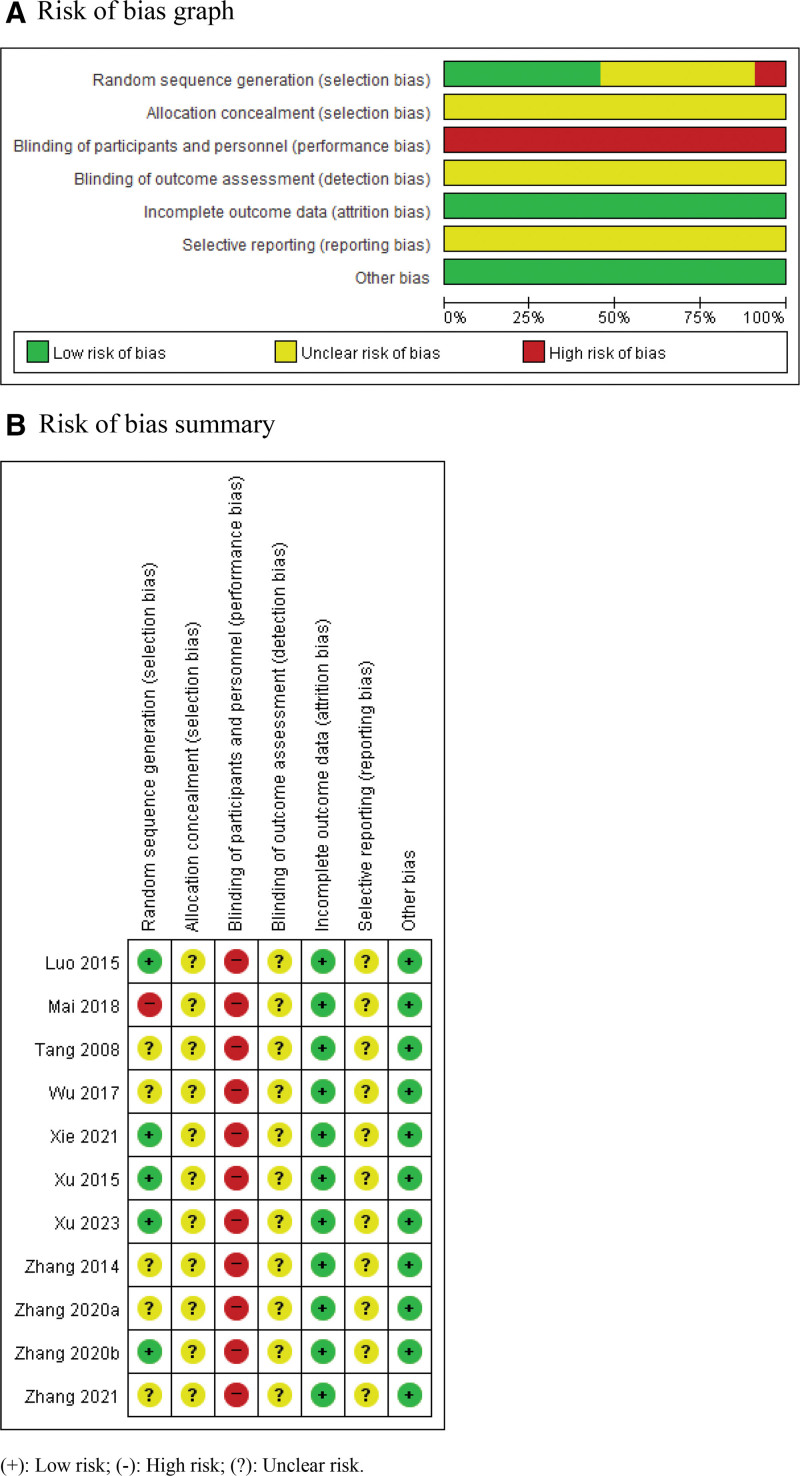
Risk of bias. (A) Risk of bias graph; (B) risk of bias summary. Risk of bias graph: review the author’s judgments about each risk of bias item presented as percentages across all included studies. Risk of bias summary: review the author’s judgments about each risk of bias item for each included study. (+): low risk of bias; (-): high risk of bias; (?): unclear risk.

ROB was assessed using the Cochrane ROB tool (Fig. 2). Five RCTs^[29,31,32,34,38]^ showed a low ROB in random sequence generation. One RCT^[30]^ was randomized according to the treatment order and was considered to have a high ROB. Five RCTs^[28,33,35–37]^ did not report random sequence generations. None of the RCTs described allocation concealment methods, anonymization of participants and personnel, or outcome assessments. None of the RCTs included dropouts or withdrawal. Incomplete outcome data were associated with a low ROB. None of the RCTs registered or published protocols, and all had an unclear ROB regarding selective outcome reporting.

### 3.4. Outcome measurement

#### 3.4.1. Total treatment effect

##### 3.4.1.1. Acupuncture versus Western medicine

One RCT^[28]^ compared the effects of acupuncture with those of Western medicine. The control group received external and intralesional injections of western medicine. This RCT showed equivalent effects between the 2 groups (External: RR 1.04, 95% CI 0.86–1.25, *P* = .69; intralesional injections: RR 0.94, 95% CI 0.83–1.07, *P* = .34).

##### 3.4.1.2. Acupuncture + plum-blossom needling versus western medicine

One RCT^[29]^ compared the effects of PBN combined with acupuncture with those of Western medicine alone. The intervention group showed favorable effects (RR 1.50, 95% CI 1.09–2.06, *P* = .01).

##### 3.4.1.3. Plum-blossom needling versus Western medicine

One RCT^[30]^ compared the effects of PBN alone with those of Western medicines. The RCT in the control group used a minoxidil tincture. Western medicine failed to show any effects (RR 1.08, 95% CI 0.71–1.64; *P* = .72).

##### 3.4.1.4. Plum-blossom needling + Western medicine versus Western medicine

**Figure 3.**
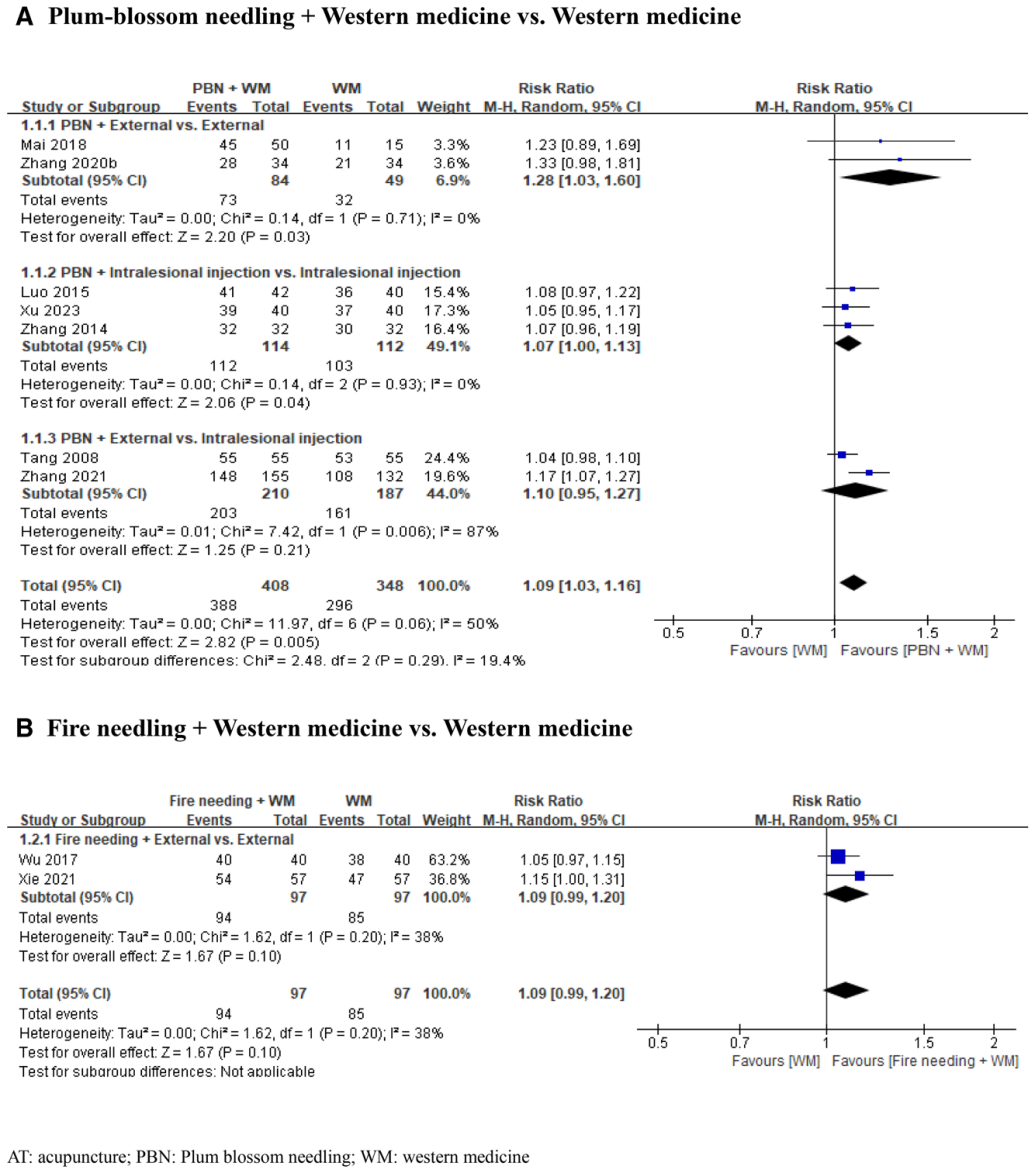
Forest plots of total treatment effect. (A) Plum-blossom needling + Western medicine versus Western medicine; (B) Fire needling + Western medicine versus Western medicine. PBN = plum-blossom needling, WM = western medicine.

Seven RCTs^[30–36]^ reported the total treatment effect. The results of the analysis showed that the symptom improvement rate was higher with PBN combined with Western medicine than with Western medicine alone (RR 1.09, 95% CI 1.03–1.16, *I*^2^ = 50%, *P* = .005, 7 RCTs, 756 participants, low CoE, Fig. 3[A]). Subgroup analysis was performed according to the type of Western medicine used.

Two RCTs^[30,31]^ tested the effects of PBN and Western medicine on the total treatment effect compared to Western medicine alone. Two RCTs failed to show an effect of PBN and Western medicine on the total treatment effects of western medicine alone. However, the meta-analysis significantly improved the total treatment effects (RR 1.28, 95% CI 1.03–1.60, *I*^2^ = 0%, *P* <.03; 2 RCTs, 133 participants).

Three RCTs^[32–34]^ examined the effects of PBN plus intralesional injection compared to intralesional injection alone in patients with AA. The RCTs showed the superior effects of PBN plus intralesional injections. The meta-analysis showed superior effects of PBN plus intralesional injection for the total treatment effect (RR 1.07, 95% CI 1.00–1.13, I^2^ = 0%, *P* = .04, 3 RCTs, 226 participants).

Two RCTs^[35,36]^ compared PBN plus external Western medicine with intralesional injections. One RCT^[36]^ showed the superior effects of PBN plus external Western medicine over intralesional injection, whereas another RCT^[35]^ showed equivalent effects between the 2 groups. The meta-analysis showed superior effects of PBN plus external Western medicine for the total treatment effect (RR 1.10, 95% CI 0.95–1.27, *I*^2^ = 87%; *P* = .21, 2 RCTs, 397 participants).

##### 3.4.1.5. Fire needling + Western medicine versus Western medicine

Two RCTs^[37,38]^ reported the total treatment effect of fire needling therapy plus external Western medicine compared with external medicine in patients with AA. Two equivalent effects were observed between the 2 groups. The meta-analysis failed to show a significant improvement in the total treatment effect (RR 1.09, 95% CI 0.99–1.20, I^2^ = 38%; *P* = .10, 2 RCTs, 194 participants, very low CoE, Fig. 3[B]).

#### 3.4.2. Hair regrowth

One RCT^[38]^ tested the effects of fire needle therapy plus external medicine on hair regrowth compared to external Western medicine in patients with AA. It showed favorable effects of fire needle therapy plus external medicine (MD -4.62, 95% CI −6.23 to −3.01, *P* <.00001).

#### 3.4.3. Rate of hair loss

No RCTs assessed the rate of hair loss as an outcome.

#### 3.4.4. Quality of life

One RCT^[34]^ examined the effects of PBN combined with intralesional injection and evaluated QoL using the Dermatology Quality of Life Index (DLQI). The results indicated a positive effect of PBN combined with intralesional injections (MD, -2.64; 95% CI −3.33 to −1.95, *P* <.00001).

#### 3.4.5. Satisfaction with hair appearance

One RCT^[33]^ tested the effects of PBN plus intralesional injection on satisfaction with hair appearance compared with Western medicine in patients with AA, and it failed to show the effects of PBN plus intralesional injection (RR 1.13, 95% CI 0.94–1.36; *P* = .19).

#### 3.4.6. Adverse events

Ten RCTs^[28,30–38]^ reported AEs. Eight RCTs^[28,30,31,33–35,37,38]^ reported AEs in the intervention group, including folliculitis, dizziness, nausea, pain, erythema on the local skin, pruritus, heat scorching, and mild redness. Ten RCTs^[28,30–38]^ reported male facial acne, skin atrophy, furuncles, irregular menses, pain, abdominal pain, distension, pruritus, erythema, scorching heat, atrophoderma, bruising, folliculitis, pigmentation, Cushing syndrome, hiccups, sleep disorders, and redness in the control group. However, no severe AEs were observed during treatment.

### 3.5. Publication bias

Publication bias could not be adequately analyzed because no meta-analysis involving more than 10 studies was performed.

### 3.6. Certainty of evidence

The CoE level for each outcome was either low or very low (Table 2).

**Table 2:** Summary of finding.

Outcomes	Anticipated absolute effects* (95% CI)		Relative effect (95% CI)	No of participants (studies)	Certainty of the evidence (GRADE)
	Risk with	Risk with			
Total treatment effect (PBN + WM vs WM)	851 per 1000	1000 per 1000 (970–1000)	RR 1.10 (1.02–1.19)	816 (7 RCTs)†	⨁⨁◯◯ Low‡
Total treatment effect (Fire needing + WM vs WM)	876 per 1000	955 per 1000 (868–1000)	RR 1.09 (0.99–1.20)	194 (2 RCTs)§	⨁◯◯◯ Very low‡,∥

GRADE Working Group grades of evidence. High certainty: we are very confident that the true effect lies close to that of the estimate of the effect. Moderate certainty: we are moderately confident in the effect estimate: the true effect is likely to be close to the estimate of the effect, but there is a possibility that it is substantially different. Low certainty: our confidence in the effect estimate is limited: the true effect may be substantially different from the estimate of the effect. Very low certainty: we have very little confidence in the effect estimate: the true effect is likely to be substantially different from the estimate of effect.

CI = confidence interval, GRADE = Grading of Recommendations Assessment Development, and Evaluation, PBN = plum-blossom needing, RCT = randomized controlled trials, RR = risk ratio, WM = western medicine.

* The risk in the intervention group (and its 95% confidence interval) is based on the assumed risk in the comparison group and the relative effect of the intervention (and its 95% CI).

† Mai (2018), Zhang (2020b), Luo (2015), Xu (2023), Zhang (2014), Tang (2008), Zhang (2021).

‡ Downgraded by 2 levels: unclear or high risk of bias.

§ Wu (2017), Xie (2021).

∥ Downgrade by one level: small sample size.

## 4. Discussion

### 4.1. Summary of the main results

This systematic review summarizes evidence regarding the efficacy of acupuncture in AA treatment. A total of 13 databases were searched for relevant studies. Finally, 11 RCTs^[28–38]^ with 1144 participants were included in this review. The reviews showed a high ROB because the studies did not report allocation concealment or anonymization of participants or researchers. Additionally, the included studies did not have any registered protocols. Therefore, acupuncture may be beneficial for AA treatment. In particular, AA symptoms, measured by the total treatment effect, showed significant improvements in the intervention group when PBN plus Western medicine was used compared with Western medicine alone.^[30–36]^ We identified 10 RCTs^[28,30–38]^ that assessed AEs. AEs occurred in both the groups; however, they were not severe.

### 4.2. Quality of evidence

The quality of the evidence was evaluated using the GRADE Pro. Patients with CoE consistently had low or very poor outcomes. This was mainly because of the high risk of bias. Some RCTs failed to include information on randomization, allocation concealment, and anonymization methods. Moreover, none of the studies were registered or had published protocols. Consequently, the evidence was reduced by 2 levels in the ROB category. In future studies, the quality of research on this topic should be improved by using rigorous research methodologies.

### 4.3. Agreements and disagreements with other studies or reviews

This review demonstrates that acupuncture treatment, alone or in combination with Western medicine, may be effective and safe for patients with AA. However, previous reviews^[17–22]^ included acupuncture combined with other traditional therapies for treating AA. In addition, some previous reviews only searched Chinese databases. Therefore, this review updates the current evidence, treatment effects, and safety of various types of acupuncture in AA treatment.

### 4.4. Implications for practice

This review shows that acupuncture, alone or in combination with Western medicine, is effective in patients with AA. The combined approach resulted in fewer AEs than Western medicine treatment alone, and the AEs were not severe. However, the results indicate the need to identify methods to reduce the pain and local redness caused by acupuncture. Therefore, future clinical studies should aim to develop a form of acupuncture with fewer AEs, which can be easily performed in patients with AA.

### 4.5. Implications for research

Our study has some limitations. First, the included studies were conducted in China and were published in Chinese. This may have influenced the potential reporting and publication biases. Further research should be conducted in countries that actively use acupuncture to treat AA. Second, the included studies lacked a detailed reporting. Registering the protocol before commencing the study could reduce reporting bias.

Moreover, the consolidated standards of reporting trials (CONSORT) guidelines^[39]^ can be used to avoid omissions. Third, this review did not identify the acupuncture points or areas used. These findings indicated that the treatment effects differed for different stimulation sites and intensities. Therefore, future studies should aim to evaluate the stimulation of acupuncture points in detail instead of simply assessing its hair-reducing effects. Fourth, the relapse rates were rarely reported in the included studies. Future studies should report long-term follow-up results. Despite these limitations, our review provides an overview of the currently available data on the effects of acupuncture on AA treatment.

The exact cause of AA in Western biomedical and complementary medicine remains unknown; however, studies have shown that acupuncture can help reduce hair loss by minimizing inflammatory attacks on the hair bulb. In addition, acupuncture may stimulate hair follicles, improve blood circulation, and enhance the local collateral circulation. Increased blood flow and nutrient delivery contribute to hair growth. Moreover, reducing stress and anxiety can positively affect growth. We hope that this study provides a reference for setting the direction for clinical treatment of AA using acupuncture and that large-scale follow-up studies and clinical practice guidelines applicable to patients will facilitate the development of this treatment modality.

## 5. Conclusions

This review summarizes the current evidence regarding the effectiveness of acupuncture in AA treatment. Combining acupuncture with western medicine is safer and more effective than acupuncture alone. However, evidence is limited due to the small number of studies and high ROB. Therefore, it is crucial to conduct well-designed RCTs to gather robust evidence regarding the effectiveness of this intervention. Further rigorous RCTs are needed to address the limitations of current evidence.

## Acknowledgments

The author would like to thank Editage (www.editage.co.kr) for English language editing.

## Author contributions

**Conceptualization:** Hye Won Lee, Myeong Soo Lee.

**Data curation:** Ji Hee Jun, Hye Won Lee.

**Formal analysis:** Ji Hee Jun.

**Funding acquisition:** Myeong Soo Lee.

**Investigation:** Ji Hee Jun, Tae-Young Choi.

**Methodology:** Ji Hee Jun, Tae-Young Choi.

**Project administration:** Ji Hee Jun.

**Writing – original draft:** Ji Hee Jun, Tae-Young Choi, Myeong Soo Lee.

**Writing – review & editing:** Ji Hee Jun, Hye Won Lee, Myeong Soo Lee.

## Supplementary Material


